# Supplementary material for: Efficacy for Whitlockite for Augmenting Spinal Fusion
Source: Int J Mol Sci. 2021 Nov 28;22(23):12875. doi: 10.3390/ijms222312875 (PMC8657587; doi:10.3390/ijms222312875)
Supplement: Supplementary file 1 [file ijms-22-12875-s001.zip › ijms-1467336-supplementary.pdf]

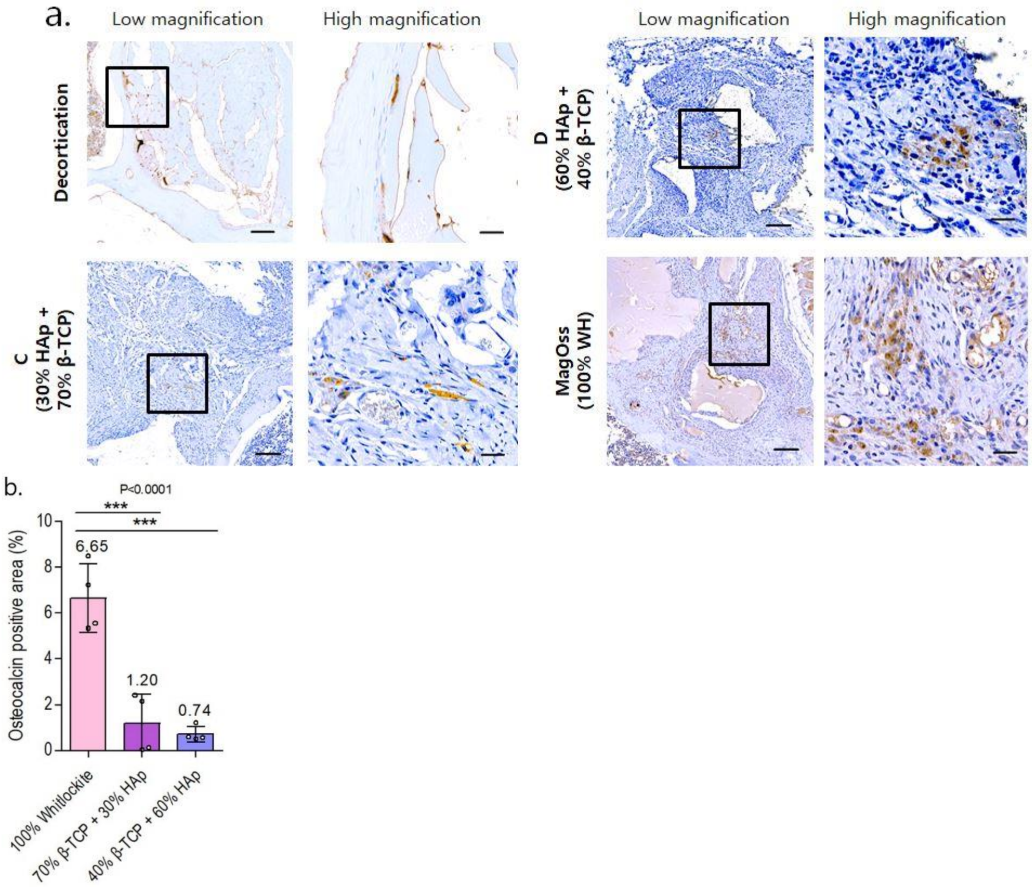

Figure S1. Immnunohistochemical analysis of bone regeneration in a spinal fusion model using staining of osteocalcin. (**a**) Images of cross-sections from each group (scale bar = 100 μm, 20 μm). (**b**) Quantitative measurements of osteocalcin-positive area (* $p < 0.05$, ** $p < 0.001$, *** $p < 0.0001$). Data were expressed as the mean ± SE (decortication, C, D, MagOss: n = 4).

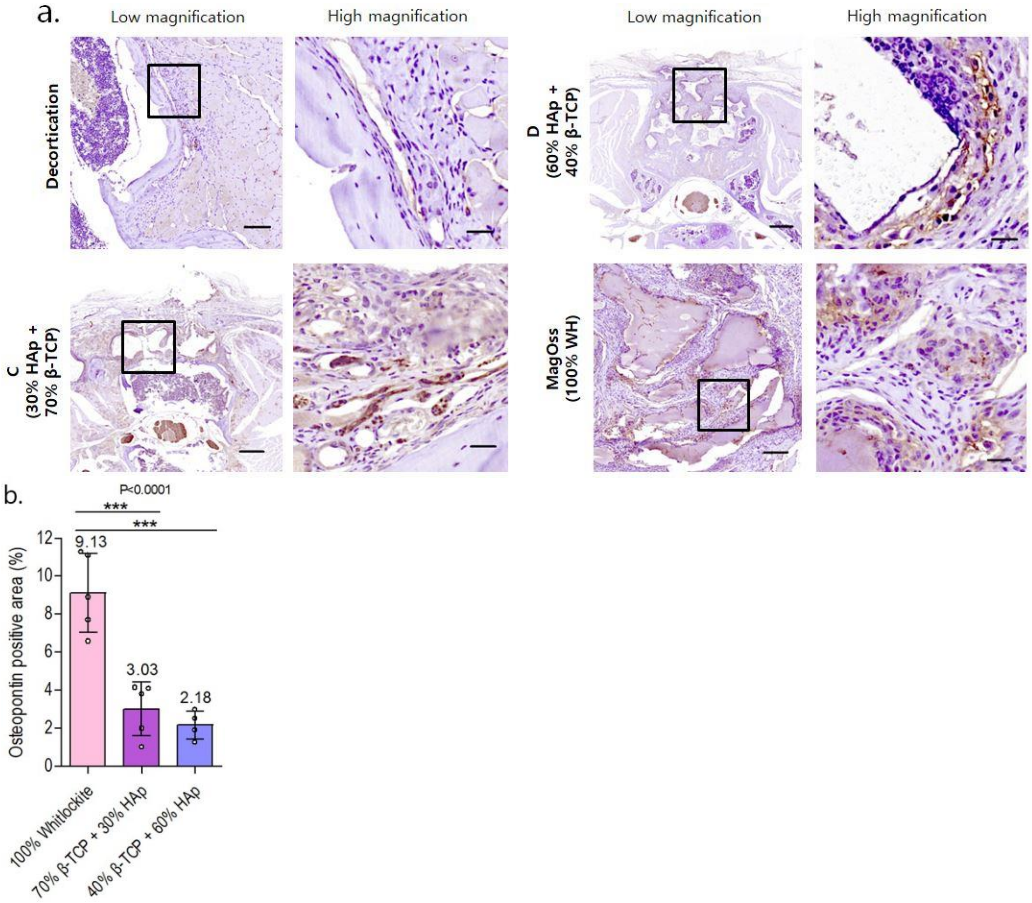

Figure S2. Immnunohistochemical analysis of bone regeneration in a spinal fusion model using staining of osteopontin. (**a**) Images of cross-sections from each group (scale bar = 100 μm, 20 μm). (**b**) Quantitative measurements of osteopontin positive area (* $p < 0.05$, ** $p < 0.001$, *** $p < 0.0001$). Data were expressed as the mean ± SE (decortication, C, MagOss: n = 5, D: n=4).

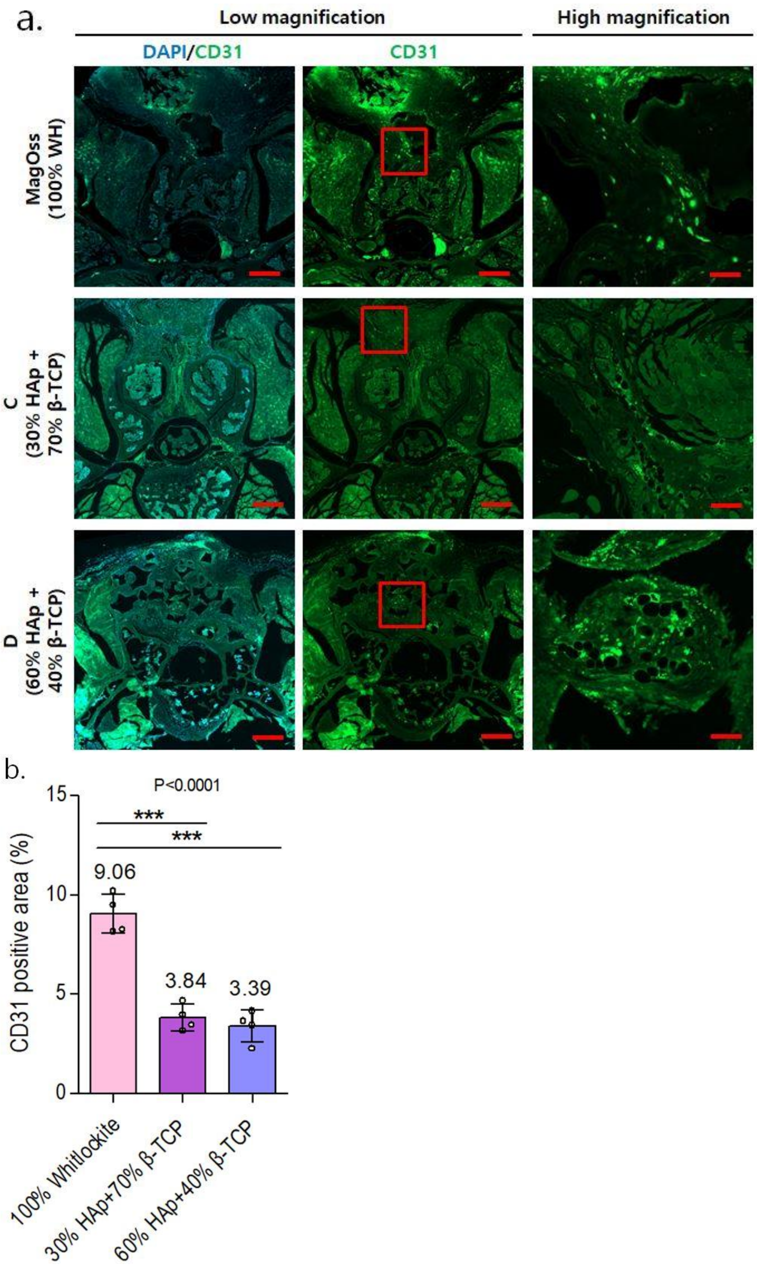

Figure S3. Immnunohistochemical analysis of bone regeneration in a spinal fusion model using staining of CD31. (**a**) Images of cross-sections from each group (scale bar = 500 μm, 100 μm). (**b**) Quantitative measurements of CD31-positive area (* $p < 0.05$, ** $p < 0.001$, *** $p < 0.0001$). Data were expressed as the mean ± SE (decortication, C, D, MagOss: n = 4).
